# A case of pleomorphic adenomas in the scrotum

**DOI:** 10.1007/s11255-023-03690-2

**Published:** 2023-08-26

**Authors:** Yong Quan, Binghang Tang

**Affiliations:** Department of Radiology, The People’s Hospital of Zhongshan, Zhongshan, 528403 China

Editor,

## Introduction

Pleomorphic adenoma (PA), also known as mixed tumor, is the most common tumor in the salivary gland, accounting for 60–70% of all salivary gland tumors [1]. According to the histological and biological characteristics of PA, the World Health Organization defines it as a borderline tumor. PA in the scrotum are rare, and a case of scrotal PA is reported here.

## Case presentation

**Fig. 1:**
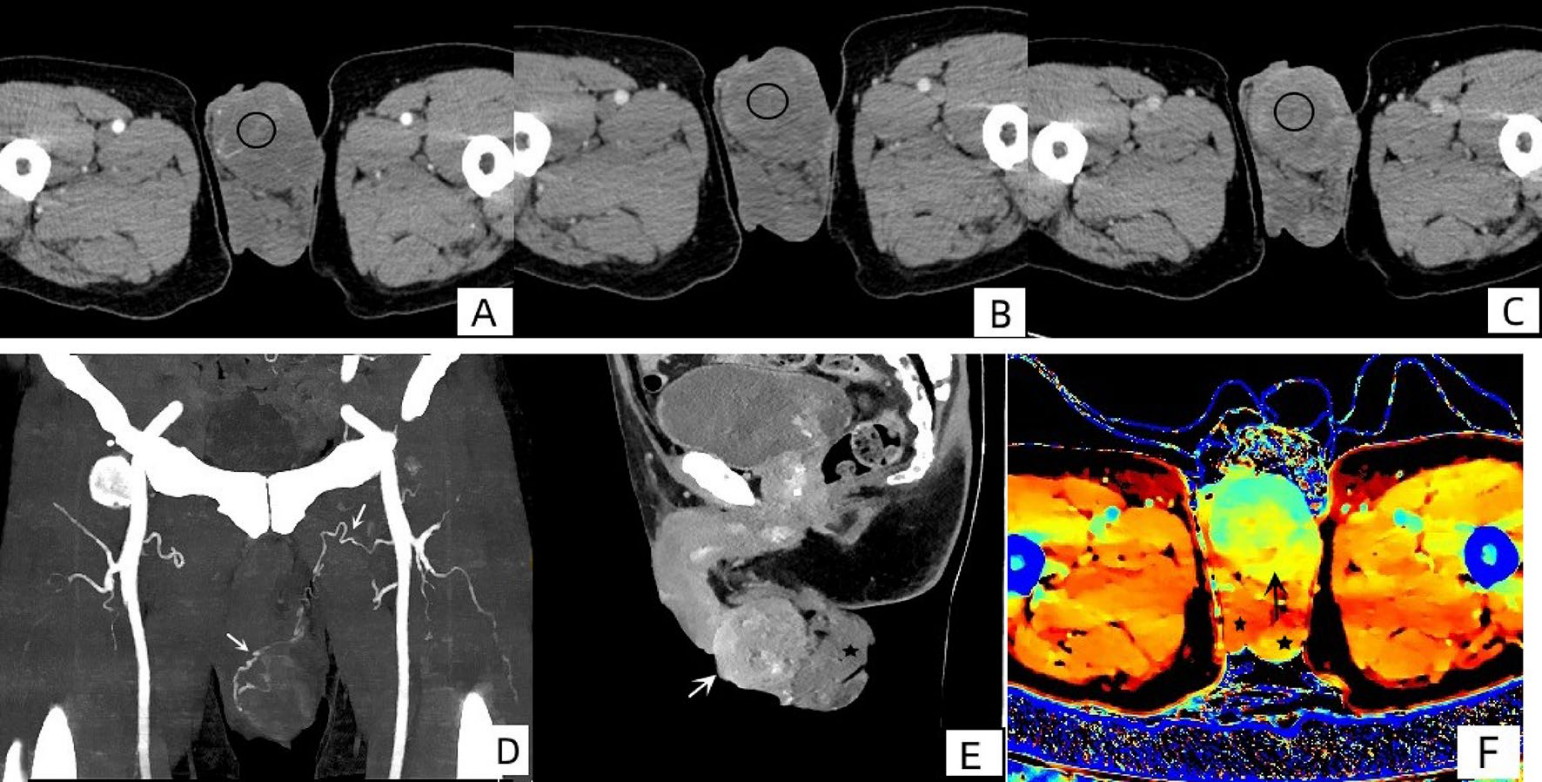
Dynamic enhanced scanning was performed in three phases, and the CT values of the mass during arterial phase (**A**), venous phase (**B**) and delayed phase (**C**) were 15, 33 and 48 HU, respectively. **D** A supply artery (white arrow) originating from the left femoral artery. **E** Delayed phase reconstruction shows uneven enhancement of the lesion (white arrow), with the left testicle (black star) in the background. **F** Z-effective image shows the mass (black arrow) and the testicle behind it (black star)

The patient was a 70-year-old male who had a left scrotal mass for more than 10 years. The lesion gradually increased in size, was hard in texture, and had no obvious tenderness upon palpation. The scrotal mass showed skin rupture and bleeding, which was difficult to stop. CT examination showed a solid soft tissue mass in the left scrotum, measuring about 55 mm × 51 mm, with an unclear border. The mass gradually strengthened, with delayed enhancement as the main feature. The CT values of the mass during arterial phase, venous phase, and delayed phase were 15, 33 and 48 Hounsfield units (HU), respectively (Fig. 1A–C). Vascular reconstruction showed a supply artery from the left femoral artery (Fig. 1D). The bilateral testes were normal in size, density, and enhancement (Fig. 1E, F). Bilateral hydrocele was present, and there were no obvious enlarged lymph nodes in the bilateral inguinal region.

**Fig. 2:**
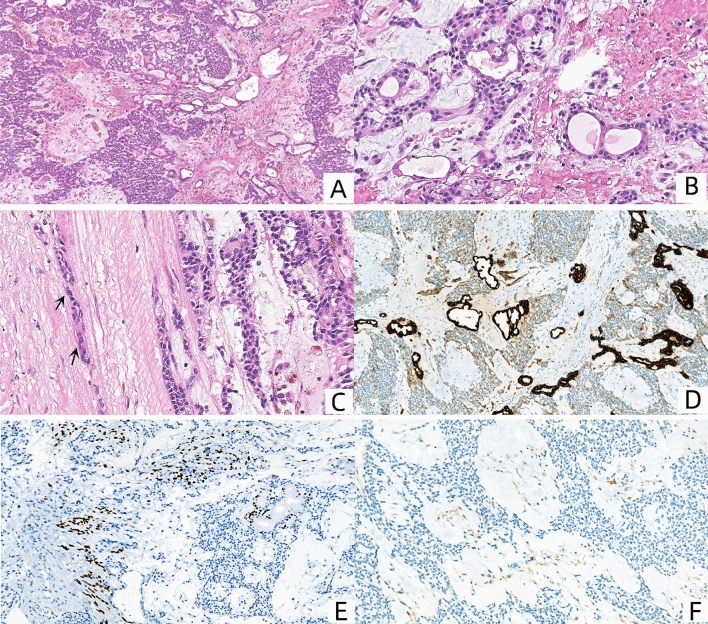
Histological and immunohistochemical features. **A** Low-power view shows that the tumor composed of ductal epithelial cells and myoepithelial cells with mucinous matrix. HE, ×10. **B** Medium-power view shows epithelial cells and myoepithelial cells within the tumor. No atypical features are seen. HE, ×40. **C** Medium-power view shows tumor cells invading the capsule. HE, ×40. **D** CK7 shows strong positivity in ductal epithelial cells. HE, ×10. **E** p63 shows positivity in some myoepithelial cells. HE, ×20. **F** Desmin is negative in any cell type of this tumor. HE, ×20

The patient underwent left scrotal mass resection and left testicular spermatic cord resection after completing relevant preoperative examinations. During the operation, the left scrotal skin and subcutaneous mass were found to be ulcerated and bleeding actively. The mass was locally adhered to the bottom of the left testis, and the left spermatic vein was tortuous. The left spermatic cord was gradually separated, and the mass, left testis, epididymis, and spermatic cord were completely removed. Histological examination showed that the tumor cells were arranged in a solid and glandular structure in a mucinous background. The cells were oval in shape, of uniform size, and the cytoplasm was acidophilic (Fig. 2A, B). There was local invasion of the tumor capsule (Fig. 2C). The testicular section was grayish-yellow and solid in nature, without any visible tumor. The epididymis was approximately 35 × 10 × 4mm in size, solid in nature, and without any obvious tumor. The spermatic cord was 55mm long, and there was no obvious tumor. The results of immunohistochemical staining showed that pan-cytokeratin (CK), CK7 (Fig. 2D) and CK5/6 labeled both epithelial and myoepithelial cells, while vimentin was positive in myoepithelial and stromal cells but negative in epithelial cells, p63 (Fig. 2E) shows positivity in some myoepithelial cells. SALL4 was only expressed positively in epithelial cells. SMA, Desmin (Fig. 2F), AFP, and PLAP were negative in any cell type of this tumor, which could be used to differentiate between smooth muscle and germ cell tumors. MyoD1 and CD117 were negative. Combined with immunohistochemistry, the final diagnosis was a left scrotal pleomorphic adenoma.

## Discussion

Pleomorphic adenoma is composed of epithelial and myoepithelial cells, and its tumor tissue contains epithelium, mucus, and cartilage-like components. It mostly occurs in the major salivary gland, but can also occur in the small salivary glands of the oral cavity, nasal cavity, pharynx, trachea, skin, breast and prostate [1–3]. Terada et al. reported the first case of pleomorphic adenomas in the spermatic cord, in a 59-year-old male patient. As the myoepithelial cells are free from the spermatic cord, and the spermatic cord is composed of urothelium, stromal cells, vasculatures, nerves, ganglion cells, and smooth muscle cells, the authors believe that spermatic cord PA is derived from the myoepithelial differentiation of urothelial cells [3].

Chondroid syringoma, also known as skin pleomorphic adenoma, originates from the sweat glands, and the reported incidence is about 0.01–0.098% [4]. Michael et al. reported three cases of skin pleomorphic adenoma, two in the inner corner of the eye and one in the eyebrow area [5]. The patient in our case had a long history, and the scrotal skin was ulcerated locally. During the operation, the pathological diagnosis of multiple pleomorphic adenomas was clear, and there was no obvious tumor invasion of the testis and spermatic cord, suggesting that it may have originated from the sweat glands of the scrotal skin. The imaging findings of this case were non-specific, and differential diagnosis with leiomyoma, rhabdomyosarcoma, or germ cell tumors should be considered clinically.

PA is a borderline tumor that usually presents as a painless, persistent mass. Patients with a long course or recurrent PA have a possibility of malignant transformation to malignant pleomorphic adenoma (MPA), and radical surgical resection is currently the preferred treatment for this disease. In this case, the patient had a long history, and the tumor capsule was invaded under the microscope, indicating the need for postoperative follow-up. Six months after scrotal mass resection, there was no recurrence in the patient at the time of writing this report.

## Data Availability

The authors confirm that the data supporting the findings of this study are available within the article.
